# Epistasis between antibiotic resistance mutations drives the evolution of extensively drug-resistant tuberculosis

**DOI:** 10.1093/emph/eot003

**Published:** 2013-03-08

**Authors:** Sònia Borrell, Youjin Teo, Federica Giardina, Elizabeth M. Streicher, Marisa Klopper, Julia Feldmann, Borna Müller, Tommie C. Victor, Sebastien Gagneux

**Affiliations:** ^1^Department of Medical Parasitology and Infection Biology, Swiss Tropical and Public Health Institute, 4002 Basel, Switzerland; ^2^University of Basel, 4003 Basel, Switzerland and ^3^DST/NRF Centre of Excellence for Biomedical Tuberculosis Research/MRC Centre for Molecular and Cellular Biology, Division of Molecular Biology and Human Genetics, Faculty of Health Sciences, Stellenbosch University, 7505 Cape Town, South Africa

**Keywords:** microbiology, antimicrobial, epidemiology, infection

## Abstract

**Background and objectives:** Multidrug resistant (MDR) bacteria are a growing threat to global health. Studies focusing on single antibiotics have shown that drug resistance is often associated with a fitness cost in the absence of drug. However, little is known about the fitness cost associated with resistance to multiple antibiotics.

**Methodology:** We used *Mycobacterium smegmatis* as a model for human tuberculosis (TB) and an *in vitro* competitive fitness assay to explore the combined fitness effects and interaction between mutations conferring resistance to rifampicin (RIF) and ofloxacin (OFX); two of the most important first- and second-line anti-TB drugs, respectively.

**Results:** We found that 4 out of 17 *M. smegmatis* mutants (24%) resistant to RIF and OFX showed a statistically significantly higher or lower competitive fitness than expected when assuming a multiplicative model of fitness effects of each individual mutation. Moreover, 6 out of the 17 double drug-resistant mutants (35%) had a significantly higher fitness than at least one of the corresponding single drug-resistant mutants. The particular combinations of resistance mutations associated with no fitness deficit in *M. smegmatis* were the most frequent among 151 clinical isolates of MDR and extensively drug-resistant (XDR) *Mycobacterium tuberculosis* from South Africa.

**Conclusions and implications:** Our results suggest that epistasis between drug resistance mutations in mycobacteria can lead to MDR strains with no fitness deficit, and that these strains are positively selected in settings with a high burden of drug-resistant TB. Taken together, our findings support a role for epistasis in the evolution and epidemiology of MDR- and XDR-TB.

## Abstract (precis)

The authors show that some mycobacteria carrying mutations conferring resistance to two antibiotics have a higher competitive fitness than corresponding strains carrying only one of these mutations. Moreover, the double-resistant strains exhibiting the highest competitive fitness in the laboratory are overrepresented in clinical settings with a high burden of extensively drug-resistant tuberculosis.



## BACKGROUND AND OBJECTIVES

Epistasis refers to the phenomenon where the phenotypic effect of one mutation differs depending on the presence of another mutation [1]. The importance of epistasis for our understanding of biology is increasingly recognized; it has been implicated in many processes, ranging from pathway organization, the evolution of sexual reproduction, mutational load, and genomic complexity, to speciation and the origin of life [2]. Moreover, recent studies have reported a role for epistasis in the evolution of antibiotic resistance [3–6]. Multidrug-resistant (MDR) bacteria are emerging worldwide, in some cases leading to incurable disease. Although new antibiotics are urgently needed, a better understanding of the forces that lead to the emergence of drug resistance would help prolong the lifespan of existing drugs.

Studies in various bacterial species have shown that the acquisition of antibiotic resistance often imposes a physiological cost on the bacteria in absence of the drug [7–9]. However, some drug resistance conferring mutations have been associated with low or no fitness cost, and compensatory evolution can mitigate some of the initial fitness defects associated with particular drug resistance conferring mutations [10]. Most of these studies have focussed on resistance to a single drug. Given the public health threat posed by MDR bacteria, there is a need to understand the factors that influence the emergence of resistance to multiple drugs.

Recent studies in model organisms have shown that mutations conferring resistance to different drugs can interact epistatically. A study in *Pseudomonas aeruginosa* found that the relative fitness of certain strains resistant to streptomycin and rifampicin (RIF) [4,6] was lower than expected based on the fitness of the corresponding single-resistant mutants. Similarly, a study in *Escherichia coli* [3] showed that strains resistant to two drugs can have a higher fitness than strains resistant to only one drug; a phenomenon referred to as ‘sign epistasis’ [11]. However, whether such epistatic interactions play any role in the emergence and spread of MDR bacteria in clinical settings has not been determined.

Multidrug resistance is a particular problem in human tuberculosis (TB) [12]. Recent surveillance data showed the highest rates of resistance ever documented with some Eastern European countries reporting up to 50% of TB cases as MDR [13]. In *Mycobacterium tuberculosis*, the main causative agent of human TB, drug resistance is chromosomally encoded and results from *de novo* acquisition of mutations in particular genes [14]. These mutations are acquired sequentially, giving rise to MDR and extensively drug-resistant (XDR) strains [15,16]. MDR-TB is defined as strains resistant to at least RIF and isoniazid, the two most important first-line anti-TB drugs. XDR-TB is caused by strains that, in addition to being MDR, are also resistant to ofloxacin (OFX), or any other fluoroquinolone, and to at least one of the injectable second-line drugs [17].

In this study, we used *Mycobacterium smegmatis* as a model for *M. tuberculosis* to investigate putative epistatic interactions between mutations conferring resistance to RIF and OFX, two of the most widely used first- and second-line anti-TB drugs, respectively. *M*. *smegmatis* is used widely in the TB research community because it is non-pathogenic, in contrast to *M. tuberculosis*, which requires biosafety-level 3 containment. Moreover, *M. smegmatis* forms visible colonies in 2–3 days, compared with 3–4 weeks for *M. tuberculosis*. We then compared our experimental data generated with *M. smegmatis* to the clinical frequency of particular combinations of RIF and OFX resistance conferring mutations in a panel of MDR and XDR *M. tuberculosis* clinical strains from South Africa.

## METHODOLOGY

### Bacterial strains and growing conditions

All strains used for the competitive fitness experiments were derived from the wild-type *M. smegmatis* strain mc^2^155. Bacteria were grown in Middlebrook 7H9 broth supplemented with ADC or on Middlebrook 7H11 agar plates supplemented with OADC. The culture tubes were incubated in standard conditions and the optical density (OD_600_) was recorded daily to measure the growth.

### Selection of single- and double-resistant *M. smegmatis* mutants

Independent RIF- and OFX-resistant *M. smegmatis* single mutants were isolated as follows. A starting culture of *M. smegmatis* mc^2^155 was prepared from wild-type *M. smegmatis* and adjusted to ∼300 bacilli/ml (OD_600_ ∼0.01). Ten milliliter of culture was transferred into 14 individual 50 ml falcon tubes. When the bacteria reached end of log-phase (OD_600_ ∼3.00), the cultures were concentrated by centrifugation at 1500 rpm for 5 min, the supernatant discarded, and the bacteria resuspended in 500 μl Middlebrook 7H9 media. This concentrated bacterial culture was plated onto Middlebrook 7H11 media containing 200 μg RIF/ml for the isolation of RIF-resistant colonies, and 2 μg OFX/ml for the isolation of OFX-resistant colonies. The plates were incubated for 3–5 days at 37°C until colonies became visible. One colony from each plate was picked and sub-cultured in antibiotic-free Middelbrook 7H9 broth. For the isolation of double-resistant mutants, different *rpoB-* and *gyrA*-mutants were used to generate different combinations of mutations conferring resistance to both antibiotics. Some double-resistant mutants were selected by plating on Middlebrook 7H11-OADC media containing both 200 µg/ml of RIF and 2 µg/ml of OFX.

### Mutation identification

The main target genes for resistance to RIF and OFX are *rpoB* and *gyrA*, respectively. To detect the relevant drug resistance conferring mutations, the *rpoB* and *gyrA* genes were amplified by PCR using DNA extracted from the single- and the double-resistant mutants. The primers used to amplify the portion of the *rpoB* gene encoding the main set of mutations conferring resistance to RIF were 5′-GGA CGT GGA GGC GAT CAC ACC-3′. For amplification of the *gyrA* gene, the primers 5′-CAT GAG CGT GAT CGT GGG CCG-3′ and 5′-CAG AAC CGT GGG CTC CTG CAC-3′ were used. The same primers were used for direct DNA sequencing from the PCR product.

### Fitness assay and calculation of fitness ratio

The *rpoB-*, *gyrA-* and *rpoB–gyrA*-mutants were competed against the wild-type antibiotic-susceptible strain in antibiotic-free Middlebrook 7H9 media. A total of 100 CFU of bacteria/ml were inoculated in 10 ml of Middlebrook 7H9 media in a 1:1 ratio. For each wild-type-mutant pair, between four and eight replicate competition assays were performed. At the start of the experiment (*t* = 0 h), 50 μl from each competition culture was plated on both antibiotic-free- and antibiotic-containing Middlebrook 7H11 plates in triplicates to estimate the baseline CFU counts. The competition cultures were incubated at standard conditions on a shaking incubator at 100 rpm, and the optical densities (OD_600_) were recorded daily. After 72 h, the same competition cultures were diluted 10^5^- to 10^6^-fold and plated on both selective and non-selective Middlebrook 7H11 media to obtain the endpoint CFU counts. For both competing strains, the Malthusian parameters were calculated by taking the natural log of the endpoint CFU over the baseline CFU [7]. The mean CFU count of the three replicates was used for the calculation of the relative competitive fitness. This gave the Malthusian parameters (*m*_s_ and *m*_r_) for both strains, which correspond to the number of doublings (generations) that each strain went through during the observed time period. Finally, the relative fitness of the drug-resistant strain relative to the wild-type was determined using *W*_rs_ = *m*_r_/*m*_s_ [7]. Shapiro–Wilk test evidenced the normality of the fitness data (*P* = 0.3). Student’s *t*-test was used to detect differences in the mean fitness and the limit for statistical significance was set at *P* = 0.05. Test statistics and estimates were based on 1000 bootstrap replicates. Statistical analysis was performed with STATA SE/10.

### Measuring epistasis

To explore putative genetic interactions between drug resistance mutations, pairwise epistasis (*ε*) was measured assuming a multiplicative model in which *ε* = *W*_AB_*W*_ab_ − *W*_Ab_*W*_aB_, where *W*_ab_ is the fitness of the clone carrying alleles *a* and *b,* and capital letters represent the wild-type sensitive alleles [3]. Following this model, values of *ε* > 0.0 indicate that the fitness of the double mutant is higher than expected based on the fitness values of the individual single mutants. Similarly, values of *ε* < 0.0 indicate that the fitness of the double mutant is lower than expected based on the fitness values of the individual single mutants. We tested the normality of the epistasis data with a Shapiro–Wilk test. To test whether epistasis values were significantly different from zero, we used the error-propagation method described by Trindade *et al.* [3]. We considered that alleles *a* and *b* showed significant epistasis whenever the calculated error was smaller than the average value of *ε* (Fig. 3).

To detect the presence of sign epistasis, we performed pairwise comparisons between the fitness of each double-resistant mutant and the corresponding single-resistant mutants using a one-sided bootstrap Student’s *t*-test with 1000 replicates (Fig. 5). The combined *P*-values were obtained using Fisher’s method.

### Clinical frequency of *rpoB* and *gyrA* mutation combinations in *M. tuberculosis*

A total of 151 clinical MDR- and XDR-TB *M. tuberculosis* isolates were included in this study. These were collected in the Eastern (*N* = 99) and Western Cape (*N* = 52) Provinces of South Africa between 2008–2009 and 2001–2008, respectively. RIF and OFX resistance determining regions in the *rpoB* and *gyrA* genes were analysed using standardized PCR and sequencing [18,19]. Amplification products were sequenced using an ABI 3130XL genetic analyzer, and the resulting chromatograms were analysed using Chromas software.

## RESULTS

### Fitness cost of single drug-resistant mutants

**Figure 1.**
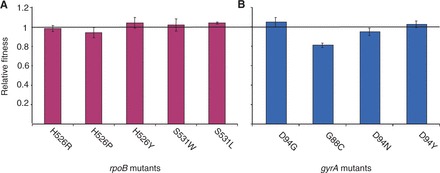
Relative fitness of *M. smegmatis* mutants resistant to a single drug compared with their pan-susceptible ancestor. Bars represent 95% confidence intervals. (**A**) Relative fitness of *rpoB* single mutants resistant to RIF. (**B**) Relative fitness of *gyrA* single mutants resistant to OFX

We first determined the relative fitness of *M. smegmatis* mutants resistant to a single drug. To this end, we selected a series of spontaneous *M. smegmatis* mutants resistant to RIF or OFX. From the RIF-selected mutants, we used five clones with *rpoB* mutations for further analysis (H526R, H526P, H526Y, S531W and S531L) (Supplementary Table S1). These mutants were competed *in vitro* against their RIF-susceptible ancestor as described previously [7]. We found that S531L, S531W and H526Y showed no difference in relative fitness compared with the ancestor (Fig. 1A), while H526R and H526P showed a significantly lower relative fitness (Bootstrap *P* = 0.02 and *P* < 0.01, respectively). Similar to previous work in *M. tuberculosis* [7], we found a strong correlation between fitness cost of *rpoB* mutations in *M. smegmatis* and the frequency of these mutations in clinical isolates of *M. tuberculosis* (Spearman’s Rank coefficient 0.9, *P* = 0.04; Supplementary Table S1). Individually, S531L and H526Y that showed no fitness cost in our *M. smegmatis* model are the most frequent RIF resistance conferring mutations in clinical settings, whereas S526P that had the lowest relative fitness of all mutants occurs only in 0.1% of clinical strains (Supplementary Table S1). We found no correlation between the spontaneous mutation frequency of *rpoB* mutations and the clinical frequency of these mutations (Supplementary Table S1).

From the OFX-selected mutants, we selected four that carried distinct *gyrA* mutations for further analysis (D94G, G88C, D94N and D94Y). *In vitro* competition against the OFX-susceptible ancestor revealed that mutants carrying D94G and D94Y had no fitness defect, while D94N and G88C had a significantly lower relative fitness (Bootstrap *P* = 0.02 and *P* < 0.01, respectively) (Fig. 1B). We compared our fitness measures with the frequency of *gyrA* mutations found in *M. tuberculosis* clinical isolates using data from a recently published review based on 1220 OFX-resistant *M. tuberculosis* isolates [20] (Supplementary Table S2). Similar to our findings with RIF-resistant mutants, we found that mutations at codon position 94 of *gyrA*, which showed overall the highest *in vitro* fitness in *M. smegmatis*, were the most common mutations in *M. tuberculosis* clinical strains. By contrast, *gyrA* G88C that had the lowest fitness is only rarely (1.6%) found in clinical settings (Supplementary Table S2). In contrast to the *rpoB* mutations, mutations at codon position 94 of *gyrA* were also the most frequent during the *in vitro* selection (Supplementary Table S2).

### Evidence for epistasis between *rpoB and gyrA* mutations

**Figure 2.**
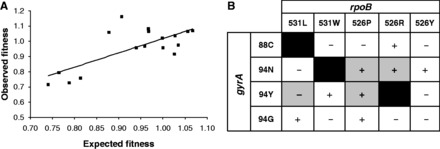
Evidence of epistasis between mutations conferring resistance to RIF and OFX. (**A**) Relationship between observed and expected multiplicative fitness for the 17 double-resistant mutants (data point above/below the bar). The solid line represents the null hypothesis of multiplicative fitness effects. Deviations from this line arise as a consequence of epistatic fitness effects. (**B**) Allelic combination analysed and the corresponding sign of epistasis. The grey squares correspond to the pairs of mutations showing statistically significant epistasis

To test for possible epistatic interactions between mutations conferring RIF and OFX resistance, we selected for spontaneous mutants resistant to both drugs. These double mutants harbouring a mutation in *rpoB* and *gyrA* were selected starting from the available single drug-resistant mutants. A total of 17 *rpoB–gyrA* double mutants were generated out of the 20 possible combinations (Supplementary Table S3). The relative fitness of the double mutants was determined by standard competition assays against the pan-susceptible ancestor strain and compared with the fitness of the corresponding single-resistant mutants. We compared the observed fitness of each double mutant with the expected fitness assuming no epistasis based on a multiplicative model (Fig. 2, see ‘Methodology’ section for details). We found that in 11/17 (65%) of the double mutants, the observed fitness was different from the expected, suggesting either negative or positive epistasis between particular RIF and OFX resistance conferring mutations (Fig. 2A).

To measure epistasis quantitatively, we measured pairwise epistasis (*ε*) between all the different single-mutant pairs we had fitness data for, assuming a multiplicative model (Supplementary Table S4); positive and negative values of *ε* indicate positive or negative epistasis, respectively [3]. Overall, the *ε-*values across all mutant pairs followed a normal distribution (Shapiro–Wilk, *P* = 0.062) with an average positive value of 0.027 (95% confidence interval −0.02, 0.08) (Supplementary Table S4). Four out of 17 (24%) double mutants showed statistically significant positive or negative epistasis between RIF and OFX resistance conferring mutations. Moreover as shown in Fig. 2B, these epistatic interactions were allele-specific, showing differences in the sign (i.e. positive versus negative) of the *ε-*value depending on the specific amino acid change at a particular codon position.

**Figure 3.**
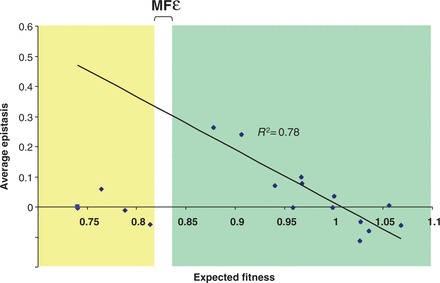
Correlation between the average expected fitness and the strength of epistasis. Average epistasis was measured as deviation from a multiplicative model of double-resistant mutant fitness scores estimated by head-to-head competition in Middlebrook 7H9 broth. MFε: minimum fitness for *ε*

Theoretical and experimental evidence predicts a correlation between the average deleterious effect of a single mutation and the strength of epistasis [21–23]. Hence, we tested whether this relationship holds for drug-resistant mycobacteria. In agreement with these predictions, we found a negative correlation between the expected fitness of our double mutants and the strength of epistasis between the respective RIF and OFX resistance conferring mutations (*R*^2 ^= 0.78; *P* < 0.001) (Fig. 3). However, this correlation was only observed above a particular threshold of expected fitness, which we refer to as ‘minimal fitness for epistasis’ (MFε). Above MFε, epistasis tended to be positive when individual mutations were costly and negative when individual mutations were beneficial [21,23]. Below MFε, the correlation was lost (*R*^2 ^= 0.07; *P* = 0.304), likely because these data points were all derived from mutants carrying the G88C mutation in *gyrA*, which was associated with a high fitness defect.

### Evidence for sign epistasis in *rpoB/gyrA* double mutants

**Figure 4.**
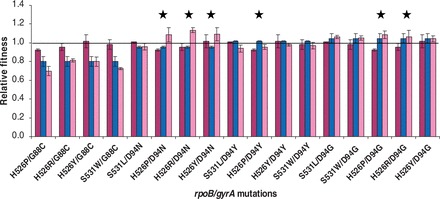
Evidence for sign epistasis between mutations conferring resistance to RIF and OFX. Sign epistasis occurs when the fitness of the double-resistant mutant (pink bar) is greater than the fitness of at least one corresponding single-resistant mutant [purple-(RIF) and blue-(OFX) bars]. The bars represent the standard deviation of the values. Double-resistant mutants with a bootstrapped *P* < 0.05 are highlighted with a star

Sign epistasis refers to the case where a particular mutation that is deleterious on its own is beneficial in the presence of another mutation [3]. In the context of drug resistance, sign epistasis occurs when the fitness of the double-resistant mutant is higher than at least one of the corresponding single-resistant mutants. We found that 6 out of 17 double mutants (35%) showed statistically significant evidence of sign epistasis (Fig. 4). In addition, the observed sign epistatis was allele specific, i.e. the epistatic effects varied according to the specific alleles of the same gene. For example, D94N in *gyrA* led to the conversion of the fitness sign in the S526P RIF-resistant background but not in the S531L RIF-resistant background (Fig. 4).

### Role of epistasis in clinical XDR-TB

**Figure 5.**
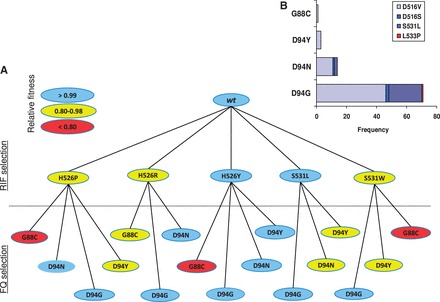
(**A**) Mutational pathway leading to *rpoB–gyrA* double mutants when a patient undergoes standard TB treatment. *RpoB* mutations are generally acquired first, followed by *gyrA* mutations. The relative fitness of the various double-resistant mutants is indicated as determined by *in vitro* competition using the *M. smegmatis* model. *wt*—drug-susceptible wild-type strain; *rpoB—*point mutations in *rpoB* conferring RIF resistance; *gyrA*—point mutations in *gyrA* conferring OFX resistance. **(B**) Frequency of *rpoB–gyrA* mutation pairs found in MDR- and XDR-TB clinical isolates from the Eastern Cape and Western Cape Provinces of South Africa (only considering pairs including *gyrA* mutants for which *M. smegmatis* fitness data were available; N = 89)

Given the evidence for epistasis between RIF and OFX resistance mutations in *M. smegmatis*, we investigated how fitness changes along the mutational pathway leading from MDR-TB to XDR-TB might be influenced by corresponding epistatic interactions in *M. tuberculosis* (Fig. 5A). In the standard treatment protocols for TB [17], RIF is an essential part of the first-line regimen for drug-susceptible disease, and OFX is a part of the second-line regimen when resistance against first-line drugs has developed. Thus, *rpoB* mutations are generally acquired first and *gyrA* mutations second. Following this trajectory, selection by RIF will occur first, and the RIF-resistant mutants that survive will exhibit heterogeneous fitness in the absence of the drug depending on their *rpoB* mutations (Fig. 1) [7,24]. At this point, MDR-TB has developed and second-line treatment is initiated. Selection for OFX resistance begins, but the fitness levels of the emerging double mutants can still be positively or negatively affected depending on which *gyrA* mutation is acquired. Our *M. smegmatis* data showed that the *gyrA* D94G mutation was associated with improved fitness in all of the double mutants, irrespective of the *rpoB* mutation (pink bars compared with purple bars in Fig. 4). This was statistically significant in two of the five corresponding double mutants tested. Hence, based on the most likely clinical scenario of moving from MDR- to XDR-TB (Fig. 5A), we would expect the *gyrA* D94G mutation to be the most commonly found mutation in XDR-TB strains, and also to be found in combination with many different *rpoB* mutations. By contrast, we would expect *gyrA* G88C, which was consistently associated with negative epistasis in our *M. smegmatis* model (Figs 3, 4 and 5A), to show the opposite trend. To test these predictions, we analysed 151 MDR- and XDR-TB clinical isolates from South Africa. Sequencing of the relevant genes revealed that 71/151 (47%) harboured *gyrA* D94G whereas G88C occurred only once (0.7%). Moreover, among the *gyrA* mutations represented in the *M. smegmatis* dataset, *gyrA* D94G was the only mutation that occurred in combination with four different *rpoB* mutations in clinical strains (Fig. 5B). Taken together, our results show that experimental fitness data generated with *M. smegmatis* can be predictive of clinical TB. Moreover, these findings support a role for epistasis in the progression of *M. tuberculosis* from MDR to XDR.

## CONCLUSION AND IMPLICATIONS

In this study, we used *M. smegmatis* as a model to show that epistasis can occur between mutations conferring resistance to RIF and OFX, which are two of the most important anti-TB drugs. Specifically, in several of the mutants resistant to both of these drugs, some of the mutations conferring resistance to one drug mitigated the negative fitness effects of some of the mutations conferring resistance to the other drug (or vice versa). Moreover, we found clear evidence of sign epistasis, showing that in some cases, the double-resistant mutants had a higher relative fitness than at least one of the corresponding single-resistant mutants. In the context of MDR, sign epistasis between different drug resistance conferring mutations represent the worst case scenario; instead of accumulating fitness defects with each additional drug resistance, MDR strains manage to increase their relative fitness by acquiring additional drug resistance determinants. One limitation of our study is that we cannot exclude the possibility that additional mutation(s) could have arisen during the selection of our mutants, which may compensate for the initial fitness defects associated with the individual resistance mutations.

More work is needed to elucidate the mechanisms involved in the interaction between mutations in *rpoB* and *gyrA*. Yet, several features make such interactions biologically plausible. *GyrA* encodes one of the subunits of DNA gyrase which is involved in the introduction of negative supercoiling to double-stranded DNA, thereby relaxing the positive supercoils that form during DNA replication [25]. *RpoB* encodes a part of the RNA polymerase and therefore important for the transcription of DNA to RNA [26]. Although these two pathways are separate [27,28], GyrA and RpoB are both involved in the fundamental flow from DNA to RNA. Intriguingly, Gupta *et al.* isolated an ‘RNA-polymerase-DNA gyrase complex’ in *M. smegmatis* that exhibited both DNA supercoiling and transcriptional activities. The authors also found that DNA gyrase inhibitors not only reduced DNA gyrase activity but also reduced transcriptional activity indicating a role of DNA gyrase in transcription [29]. Finally, it has been shown that during transcription, RNA polymerase introduces positive supercoiling ahead as it slides along its template DNA. This leads to a reduced accessibility as supercoiling increases, further supporting a potential role for DNA gyrase in transcription [25].

Our study also showed that experimental data obtained from *M. smegmatis* are relevant for our understanding of clinical TB. Not only did we observe the same drug resistance conferring mutations in *M. smegmatis* as routinely encountered in clinical strains of *M. tuberculosis*, but similar to previous studies, we found a good correlation for both RIF and OFX between the fitness cost observed *in vitro* in *M. smegmatis* mutants and the relative clinical frequency of the corresponding mutations in *M. tuberculosis* [20,24]. Our *M. smegmatis* data showed particular relevance when focusing on MDR- and XDR-TB. Based on the most probable mutational pathway leading from MDR to XDR, our *M. smegmatis* fitness data predicted particular combinations of *rpoB* and *gyrA* mutations to be more frequent than others in clinical settings. This prediction was confirmed when screening a large panel of MDR and XDR *M. tuberculosis* clinical strains from South Africa, which is one of the regions with the highest burden of XDR-TB in the world [17].

Our mutational pathway analysis also showed that in some cases, if certain mutations are acquired first, the fitness of these drug-resistant strains is permanently set at a high baseline that cannot be drastically affected regardless of the individual fitness cost associated with the second mutation. Moreover, some *gyrA* mutations can act as ‘fitness safety nets’ offering the bacteria the possibility to recover from loss of fitness caused by any of the initial *rpoB* mutations. Taken together, our results suggest that although evolution towards MDR- and XDR-TB can follow multiple trajectories, these are likely to be influenced by epistatic interactions between the particular drug resistance conferring mutations. This will constrain the particular mutational combinations to those that either increase or at least maintain fitness at a minimum level (Fig. 4). Above this minimum level of fitness, our study indicates that the strength of epistasis between *gyrA* and *rpoB* will be stronger when the individual mutations are associated with large fitness defects. Although the fitness measures reported here were generated during *in vitro* growth, *M. tuberculosis* is facing harsher environments during human infection. The fitness effects of drug resistance mutations have been shown to vary in different environments [6,30]. Hence, it would be interesting to explore how host immune pressure, oxidative and other stresses might influence epitasis between drug resistance mutations.

Our finding that a specific *gyrA* mutation (i.e. D94G; Figs 4 and 5A) can restore the fitness of strains carrying different *rpoB* mutations has implications for the development of new TB treatment regimens. So far, OFX and other fluoroquinolones have primarily been used as second-line drugs to treat MDR-TB [31]. However, because of their potential to shorten TB chemotherapy, they are currently being evaluated in the context of new first-line treatment regimens for drug-susceptible TB [32]. Our results highlight that using fluoroquinolones as first-line treatment is likely to result in the early selection of fluoroquinolone resistance conferring mutations such as D94G *gyrA* that not only confer resistance but might promote also the acquisition of additional drug resistance while maintaining bacterial fitness at an advantageous level, either through positive epistasis with mutations conferring resistance to RIF or other drugs, or by establishing a higher baseline fitness [33]. Moreover, exposure to fluoroquinolones induces the bacterial SOS response which leads to the induction of error-prone DNA polymerases, thereby increasing the bacterial mutation rate and the propensity of acquiring additional drug resistance conferring mutations [34]. Interestingly, we found that resistance mutations at codon position 94 of *gyrA* were also most frequent during *in vitro* selection, suggesting that in addition to epistatic interactions between *rpoB* and *gyrA* mutations, other mechanisms might influence the frequency of particular combinations of drug resistance mutations in clinical settings.

In conclusion, our study together with previous findings demonstrates that epistasis between different drug resistance conferring mutations occurs across several bacterial species. Although our study focused on the interaction between mutations in *rpoB* and *gyrA*, further work should explore possible similar effects in resistance to other anti-TB drugs, both existing as well as those currently under development [35] (http://www.newtbdrugs.org/pipeline.php). Three new drug candidates have shown promising results in recent clinical trials of MDR-TB treatment [32]. However, how these new compounds should best be deployed, and in what combinations, remains unclear. Our study suggests that considering putative epistasis between the relevant drug resistance conferring mutations could help optimize treatment regimens. For example, combining drugs in which the resistance conferring mutations interact negatively would reduce the probability of resistance emerging.

## SUPPLEMENTARY DATA

Supplementary data is available at *EMPH* online.

Supplementary Data
