# Supplementary material for: Epistasis between antibiotic resistance mutations drives the evolution of extensively drug-resistant tuberculosis
Source: Evol Med Public Health. 2013 Mar 8;2013(1):65–74. doi: 10.1093/emph/eot003 (PMC3868377; doi:10.1093/emph/eot003)
Supplement: Supplementary Data [file supp_2013_1_65__index.html]

Epistasis between antibiotic resistance mutations drives the evolution of extensively drug-resistant tuberculosis — Supplementary Data 

# Epistasis between antibiotic resistance mutations drives the evolution of extensively drug-resistant tuberculosis

## Supplementary Data

files

**Files in this Data Supplement:**

- Supplementary Data - pdf file
