# Supplementary material for: Epistasis between antibiotic resistance mutations drives the evolution of extensively drug-resistant tuberculosis
Source: Evol Med Public Health. 2013 Mar 8;2013(1):65–74. doi: 10.1093/emph/eot003 (PMC3868377; doi:10.1093/emph/eot003)
Supplement: Supplementary Data [file supp_eot003_Suppl_Tables_reviewed.pdf]

Suppl Table 1. Rifampicine (RIF) single-resistant mutants used in the study.

Sample name	RIF resistance					
	Conc (µg/ml)	*rpoB* SNP	Rel. Fitness	Stdev	% in Clinical TB*	Freq. *in vitro* mutants (N=15)
S164	200	S531L	1.00	0.002	54.3	6.7%
S021	200	H526Y	1.01	0.071	10.6	13.3%
S025	200	S531W	0.98	0.052	4.0	6.7%
S016	200	H526R	0.95	0.037	2.8	60.0%
S018	200	H526P	0.92	0.014	0.1	13.3%

* Based on data extracted from 840 clinical isolates with a single *rpoB* mutation summarized in ref. 24.
Spearman's rho = 0.9 p-value= 0.04

Suppl Table 2. Ofloxacine (OFX) single-resistant mutants used in the study.

Sample name	OFX resistance					
	Conc (µg/ml)	*gyrA* SNP	Rel. Fitness	Stdev	% in Clinical TB*	Freq. *in vitro* mutants (N=19)
S002	2	D94G	1.04	0.047	64.7	89.5%
S013	2	D94Y	1.01	0.004		
S004	2	D94N	0.95	0.011		
S003	2	G88C	0.8	0.051	1.6	10.5%

* Based on data extracted from 691 clinical isolates with a single *gyrA* mutation summarized in ref 20. That metanalysis was based on codons, no allele-specific information was included.

Suppl Table 3. Competitive fitness of mutants resistant to rifampicine (RIF) and ofloxacine (OFX).

Sample name	RIF-OFX resistance			
	rpoB SNP	*gyrA* SNP	Rel. Fitness	Stdev
S118	H526R	G88C	0.80	0.018
S101	H526P	G88C	0.71	0.051
S128	H526Y	G88C	0.76	0.051
S148	S531W	G88C	0.72	0.01
S114	H526R	D94N	1.16	0.025
S099	H526P	D94N	1.06	0.074
S137	H526Y	D94N	1.06	0.068
S071	S531L	D94N	0.97	0.035
S136	H526Y	D94Y	0.97	0.013
S142	S531W	D94Y	0.96	0.034
S107	H526P	D94Y	0.95	0.026
S094	S531L	D94Y	0.91	0.037
S097	H526P	D94G	1.08	0.041
S127	H526Y	D94G	1.07	0.032
S048	S531L	D94G	1.06	0.016
S138	S531W	D94G	1.03	0.022
S117	H526R	D94G	1.01	0.071

Suppl Table 4. Epistasis (ε) in mutants resistant to rifampicine (RIF) and ofloxacine (OFX).

Sample name	RIF-OFX resistance		ε	Stdev ε	95% CI *	
	rpoB SNP	*gyrA* SNP			Lower	Upper
S101	H526P	G88C	-0.004	0.070	-0.145	0.136
S118	H526R	G88C	0.059	0.060	-0.060	0.178
S128	H526Y	G88C	-0.059	0.093	-0.244	0.126
S148	S531W	G88C	-0.011	0.066	-0.143	0.120
S071	S531L	D94N	-0.001	0.037	-0.075	0.072
S099	H526P	D94N	0.263	0.075	0.112	0.414
S114	H526R	D94N	0.240	0.044	0.151	0.329
S137	H526Y	D94N	0.077	0.097	-0.117	0.270
S094	S531L	D94Y	-0.113	0.037	-0.188	-0.039
S107	H526P	D94Y	0.071	0.030	0.012	0.131
S136	H526Y	D94Y	-0.081	0.074	-0.229	0.067
S142	S531W	D94Y	0.036	0.063	-0.089	0.162
S048	S531L	D94G	0.006	0.050	-0.095	0.107
S138	S531W	D94G	-0.050	0.075	-0.200	0.099
S097	H526P	D94G	0.099	0.062	-0.025	0.223
S117	H526R	D94G	-0.003	0.093	-0.188	0.182
S127	H526Y	D94G	-0.062	0.095	-0.251	0.127

RIF; rifampicine, OFX; ofloxacine, CI; Confidence Interval
